# Microfluidic Thrombosis under Multiple Shear Rates and Antiplatelet Therapy Doses

**DOI:** 10.1371/journal.pone.0082493

**Published:** 2014-01-03

**Authors:** Melissa Li, Nathan A. Hotaling, David N. Ku, Craig R. Forest

**Affiliations:** 1 Wallace H. Coulter Department of Biomedical Engineering, Georgia Institute of Technology, Atlanta, Georgia, United States of America; 2 George W. Woodruff Department of Mechanical Engineering, Georgia Institute of Technology, Atlanta, Georgia, United States of America; University of California Irvine, United States of America

## Abstract

The mainstay of treatment for thrombosis, the formation of occlusive platelet aggregates that often lead to heart attack and stroke, is antiplatelet therapy. Antiplatelet therapy dosing and resistance are poorly understood, leading to potential incorrect and ineffective dosing. Shear rate is also suspected to play a major role in thrombosis, but instrumentation to measure its influence has been limited by flow conditions, agonist use, and non-systematic and/or non-quantitative studies.

In this work we measured occlusion times and thrombus detachment for a range of initial shear rates (500, 1500, 4000, and 10000 s^−1^) and therapy concentrations (0–2.4 µM for eptifibatide, 0–2 mM for acetyl-salicylic acid (ASA), 3.5–40 Units/L for heparin) using a microfluidic device. We also measured complete blood counts (CBC) and platelet activity using whole blood impedance aggregometry. Effects of shear rate and dose were analyzed using general linear models, logistic regressions, and Cox proportional hazards models.

Shear rates have significant effects on thrombosis/dose-response curves for all tested therapies. ASA has little effect on high shear occlusion times, even at very high doses (up to 20 times the recommended dose). Under ASA therapy, thrombi formed at high shear rates were 4 times more prone to detachment compared to those formed under control conditions. Eptifibatide reduced occlusion when controlling for shear rate and its efficacy increased with dose concentration. In contrast, the hazard of occlusion from ASA was several orders of magnitude higher than that of eptifibatide. Our results show similar dose efficacy to our low shear measurements using whole blood aggregometry. This quantitative and statistically validated study of the effects of a wide range of shear rate and antiplatelet therapy doses on occlusive thrombosis contributes to more accurate understanding of thrombosis and to models for optimizing patient treatment.

## Introduction

Thrombosis, the formation of occlusive platelet aggregates in blood, is the primary cause for the pathology of stroke and heart attack. Thrombosis can be treated with antiplatelet therapies, but these are not effective for many patients, with an estimated 5–45% still undergoing adverse cardiovascular events after treatment [1], [2] depending on the therapy used. Incorrect doses of antiplatelet therapies can have side effects including severe bleeding, gastrointestinal discomfort, and death in some cases. The majority of these cases are due to idiopathic “aspirin resistance” [3]–[6], while the use of GPIIb/IIIa inhibitors, such as eptifibatide/Integrilin®, have also contributed. The mechanisms for antiplatelet therapy resistance and guidelines for appropriate doses are poorly understood [7], [8]. Thus, instrumentation for evaluating thrombosis before and after application of antiplatelet therapy would provide valuable feedback in clinical studies and personalized patient treatment for optimizing therapies and their respective doses.

The International Society on Thrombosis and Hemostasis (ISTH) has recommended criteria for the design of such instrumentation [9]. First, flow conditions in antiplatelet therapy instrumentation should properly reproduce conditions in the vasculature, including multiple shear rates spanning physiological to pathological levels. Further, the flow environment should feature a pathologically relevant eccentric constriction, or stenosis. Within this flow environment, instrumentation should enable continuous monitoring to examine not only fully occlusive thrombosis, but also the possibility of thrombus detachment, which can lead to clinical correlates of stroke or embolism. Instrumentation should be capable of measuring the effects of multiple therapies in combination, as they are commonly prescribed. Finally, although antibody or fluorescence labeling and microscopy are very effective for identifying platelets at the early stages of platelet adhesion, the high cost and bulk of associated equipment employed in such techniques can limit use, especially at the point of care [9].

While commercial methods that have been developed have focused specifically on assaying platelet function before and after addition of therapy, they have to date shown poor efficacy in clinical trials [10], [11]. A large part of these challenges in performance has been ascribed the common use of *in-vitro* anticoagulant-agonist combinations such as citrate and ADP [9], [12] that eliminate important aspects of *in-vivo* platelet function. Furthermore, in comparison to ISTH recommendations, these commercialized methods are often only able to perform testing on a single sample and/or at a single shear rate under flow conditions–including high speed bead mixing, forced flow through a membrane, and cone-and-plate flow–that are not relevant to biological flow through vasculature and have shown inconclusive results [1], [13].

Research using traditional flow chambers has demonstrated that at certain shear rate ranges specific sets of proteins and small molecules govern thrombosis. Specifically, previous work has described three shear rate regimes: low shear “venous flow” (100–1000 s^−1^), primarily governed by fibrinogen, coagulation factors, and the GPIIb/IIIa (also known as integrin β_IIb_α_3_); “arterial flow” (1000–4000 s^−1^), primarily governed by GPIb, GPIIb/IIIa, and soluble agonists such as adenosine diphosphate (ADP) or cyclooxygenase (COX) enzymes; and “high pathologic flow” (>4000 s^−1^) commonly found in diseased, constricted, or “stenosed”, arteries, primarily governed by vWF and GPIb [9], [14]–[16]. In addition to these aforementioned shear-related binding events, additional interactions such as the GPVI association to collagen occur at all shear rates, although these bonds can be significantly enhanced at higher shear rates by the presence of vWF [16]. Many of these shear-sensitive binding factors are targets for antiplatelet therapies. For example, GPIIb/IIIa inhibitors (eptifibatide, abciximab), soluble activation inhibitors (clopidogrel, aspirin), and anti-coagulants (heparin, citrate) [9]. Thus, changes in shear rate may affect the efficacy of antiplatelet therapies at mitigating thrombosis. However, systematic characterization of shear-related changes in antiplatelet efficacy using flow chamber methods is very difficult due to their low throughput or large sample volumes.

In contrast to traditional flow chambers, the development of high throughput, low volume microfluidic methods have proven well suited for the study of platelet function for recent clinical and research applications. For example, low sample volumes have enabled techniques including genetic knockout screenings in murine models [17] and clinical assays of human blood [18]. Meanwhile, high throughput capabilities have also enabled systematic characterization of multiple sample parameters, such as simultaneous measurement of platelet function over a range of shear flow conditions [12], [17], [19], [20]. Additionally, soft lithography and micromilling fabrication techniques used have allowed local protein micropatterning of collagen to simulate focal vascular injury [20], [21] and the construction of complex and customizable flow environments to study the effects of vessel stenosis morphology thrombosis [22], respectively. Using a microfluidic system, Hosokawa et al. [19] have examined changes in platelet adhesion due to doses of platelet therapies and low, venous shear rates (240, 600 s^−1^) within non-stenotic channel geometries. Similarly, the Bioflux Live Cell Imaging system, composed of a microfluidic flow chamber coupled to a well plate and fluorescence microscopy system to examine adhesion of platelets and endothelial cells before and after addition of GPIIb/IIIa antiplatelet therapy [23]. Such high throughput and reliability require costly microscopy and cell culture equipment. While both of these groups examined changes in therapy efficacy due to shear rate and antiplatelet therapy dose, the shear range and channel geometries used were not relevant to the intended therapeutic target of higher pathological shear rates and stenotic vascular geometries.

Inter-subject and intra-subject variations in phenotypic factors including hematocrit, platelet count, and vWF can also impact measurements made from platelet function devices [1]. Recent work by Neeves *et al.* presented the largest investigation to date of microfluidic flow assays supplanted with data from subjects' complete blood counts (CBC), platelet receptor genotyping, and vWF titer assays to characterize such variations [12]. This work showed the importance of vWF plasma levels and the collagen-binding GPVI receptor in platelet surface adhesion (surface coverage <15%) using desirable small volumes (<1 mL), yet it did not characterize platelet-platelet aggregation and cohesion, rapid platelet accumulation, high shear rates, and/or thrombus stability—all factors implicated in the later stages of thrombus development and associated with a different set of binding events [9], [24], [25]. Thus, although microfluidic tools have previously shown great promise for studies of the combined effects of multiple shear rates, antiplatelet therapies, and inter-patient variations, current research has been limited by one or more of the following parameters: limited monitoring of thrombus formation from initial adhesion without later stage aggregation and detachment metrics [12], [17], [20], [23], [26], non-stenotic flow [1], [27], [28], small range of non-pathologically relevant shear rates [17], [29], non-physiologically relevant anticoagulant-agonist use [1], low-throughput and/or non-systematic studies [24], [30], and limited characterization of inter-patient CBC variation [12].

In previous work, we have described the design and application of a microfluidic system for simultaneous optical measurement of thrombosis at multiple, well-defined shear rates simultaneously in whole porcine blood without the addition of antiplatelet therapies, and presented a proof of concept for applying clinically derived pathological flow conditions to whole blood samples in a microscope-free, high throughput, microscale system [31]. We now apply this system to human blood across a range of shear rates and antiplatelet therapy concentrations to accurately, robustly, and statistically quantify metrics of platelet activity for a population. In this study, thrombosis was quantified using metrics of occlusion time, thrombus detachment likelihood, and a Cox hazard analysis to quantify the relationship between shear rate and antiplatelet therapy dose. These metrics have been chosen to quantify later-stage development, in contrast to the majority of prior thrombosis studies which focused on quantifications of platelet adhesion (most often detected as surface coverage by fluorophore labeled platelets or by minute increases in flow pressure [12], [20], [26], [28]). Furthermore, the use of Cox proportional hazards models and logistic regression allows for the direct comparative quantification of the effects of shear rate and/or therapy while controlling for all covariates; similar to methods used in clinical studies of commercial devices [11].

## Methods

### Ethics Statement

Blood samples were collected from N = 8 healthy adults with their written consent in accordance with protocol H10090 approved by the Georgia Institute of Technology Institutional Review Board.

### Sample Preparation

Blood was drawn through a 21-gauge needle into syringes pre-filled with 3.5 Units/L of unfractionated heparin (Elkins-Sinn Inc., Cherry Hill, NJ) and used within five hours of collection. In contrast to citrate, the use of heparin does not require the addition of non-physiological adenosine ADP or calcium to reactivate platelets in order to form platelet thrombus [24], [31], both of which have been found by others [32] to interfere with the function of eptifibatide. In order to ensure that addition of heparin did not inhibit platelet behavior, we also characterized occlusion times in doses of 8, 20 or 40 Units/L for subjects as shown in Table S1 in File S1 in addition to the platelet therapies listed previously.

Eptifibatide (Integrilin®, Millennium Pharmaceuticals, South San Francisco, CA) or ASA (Sigma Chemical, St. Louis, MO) was added within 20 min of sample collection. Eptifibatide was diluted in saline to a total volume of 500 µL before addition into 60 mL blood sample aliquots. Due to the short life of ASA in solution, aliquots were prepared immediately before addition to the sample by dissolving 100 mg/mL into dimethyl-sulfoxide (DMSO) (Sigma Chemical, St. Louis, MO) and then diluted into saline, as per a standard method [33]. Platelet therapies were added as discrete doses as follows: eptifibatide (0.24, 0.48, 0.72, 1.2 and 2.4 µM), or ASA (0.36, 1, 2 mM). Control (“untreated”) samples were infused with volumes of saline equivalent to those used for platelet therapies. Added volumes of either saline or antiplatelet therapy were kept below 2% of the total aliquot volume to prevent large changes in hematocrit. Therapy concentrations were chosen to reflect clinical recommendations (eptifibatide 2.4 µM, ASA 0.1 mM) based on bolus/body weight [34]–[36].

### Microfluidic System

**Figure 1:**
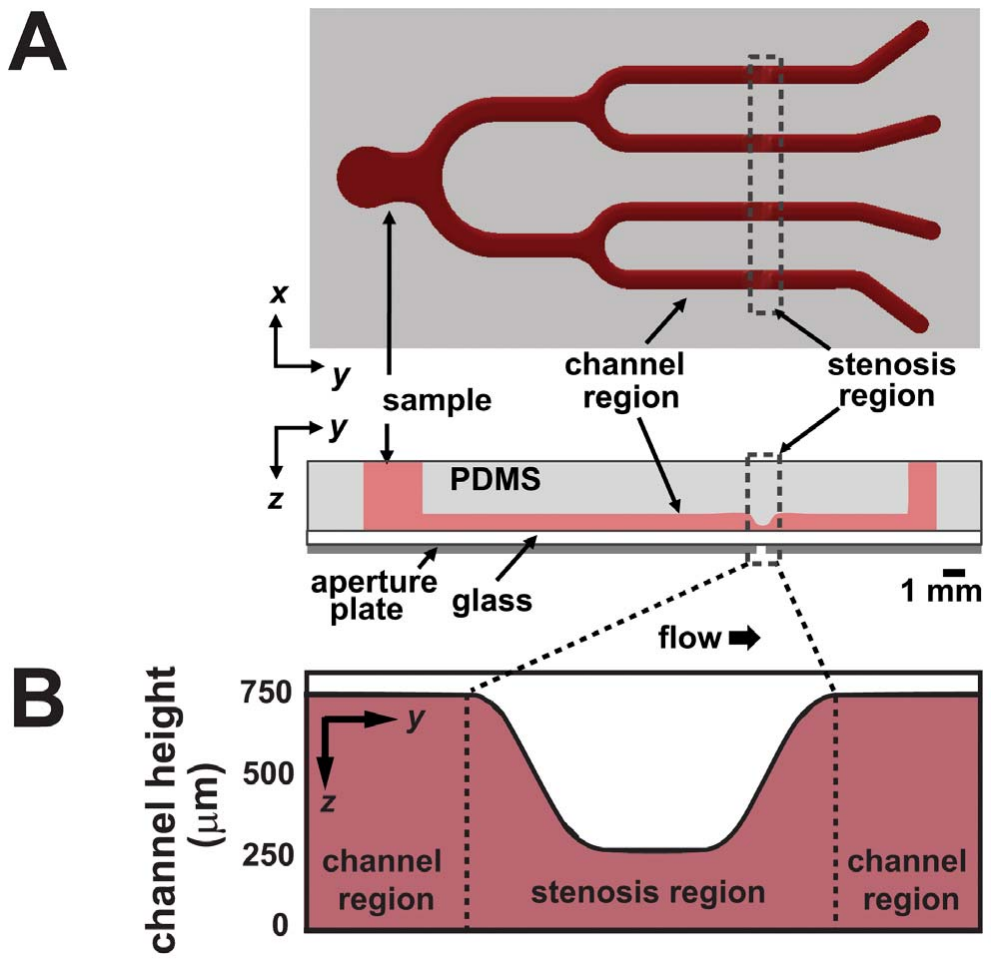
Schematic images of the microfluidic chip. The complete device is shown in (A) with detailed view of the stenosis region (B). From a single inlet, four branching channels subject the blood flow to a range of shear rates from physiological to pathological conditions. Dimensions of all four stenoses within the device are identical, with varying shear rates imposed by resistive tubing at the outlet ports.

The design and fabrication of the microfluidic system have been described in depth previously [31], [37], [38]. Briefly, devices shown in Fig. 1 were designed and validated to simultaneously address initial shear rates of 500, 1500, 4000, and 10000 s^−1^ within each of the four identical stenoses, with varying shear rates imposed by resistive tubing at the outlet ports. These shear rates were validated using finite volume fluid modeling and experimental flow rate testing. Devices were fabricated in poly-dimethylsiloxane (PDMS) and bonded to coverglass via surface plasma bonding (Harrick Plasma) to form enclosed flow channels. In order to initiate platelet adhesion, the channels were filled with fibrillar equine collagen (Chronolog Corporation, Havertown, PA) at a concentration of 100 µg/mL^−1^ overnight [16]. Before use, each device was primed with saline solution (0.85% v/v) to prevent non-specific adsorption and to remove air bubbles.

### Thrombosis Testing

Patient samples were flowed into the microfluidic system using gravity-induced pressure in elevated, open syringes at 1400 Pa. This pressure was held constant as flow emptied the syringe by optically sensing fluid height and feedback controlling syringe position with a linear actuator (Firgelli, Victoria, BC).

For each aliquot, four shear rates were run simultaneously within a single trial while platelet aggregation to occlusion was measured by four weighing scales (Ohaus, Parsipanny, NJ) at the outlet of each channel with concurrent microscopy of the stenosis regions acquired and processed using Labview (National Instruments, Austin, TX) and Matlab (Mathworks, Natick, MA), respectively. A trial, T, is defined as a single experiment with one new microfluidic device comprising four channel runs, each of a different shear rate. From N subjects, up to S samples were collected and run in T trials, each with C = 4 channel runs. Our previous studies have shown good agreement on thrombosis detection between flow rate and microscopy [31]. Occlusion time, *t_occlusion_*, was defined as the time at which a moving average flow rate (with 10 second integration time) fell below a threshold of 0.1 µL/s. Channel runs were considered “non-occluded” if the sample was exhausted during the course of the trial (e.g., 40 min) or if occlusion did not occur in less than one hour. Testing repeatability measures of intra-subject variation were derived from T = 5 repeated trials from a single subject, and inter-subject variation were derived from T = 1 trials in each of N = 7 different subjects. “Thrombus detachment” was defined by a resumption of flow above the threshold after the occlusion time. Thrombus detachment was observed concurrently using microscopy. We measured occlusion times and thrombus detachment likelihood for a range of initial shear rates (500, 1500, 4000, and 10000 s^−1^) and therapy concentrations (0–2.4 µM for eptifibatide, 0–2 mM for ASA, 3.5–40 Units/L for heparin).

#### Low Shear Impedance Aggregometry

Additional 2 mL sample aliquots were taken from patients concurrently. Of these, approximately 0.5 mL was diluted in 0.5 mL saline was used to measuring platelet activity using a Whole Blood Aggregometer (WBA) commercial instrument (Chronolog Corporation, Havertown, PA) according to manufacturer instructions. Briefly, the WBA heats and stirs the 1 mL diluted sample with a chemical agonist to induce platelets to adhere to two electrodes within a polystyrene cuvette, which causes the impedance between the electrodes to increase temporally. In these experiments, sample activity was induced using an agonist of 4 µL of collagen, stirring rate of 1200 rpm, and 6 min measurement period. As impedance increases with time, the integral of the impedance function (area under the curve, or *AUC*), serves as the primary metric for platelet activity, according to the manufacturer's directions.

### Complete Blood Count (CBC)

From the remaining volume of the 2 mL sample aliquot, approximately 0.7 mL was used for complete blood count (CBC) measurements from a Cell-Dyn Ruby (Abbott Diagnostics, Abbott Park, IL) according to manufacturer instructions. Parameters evaluated from these blood counts were: platelet count, mean platelet volume, white blood cell count, lymphocyte count, hematocrit, red blood cell count, hemoglobin concentration, and mean corpuscular volume (the volume of each red blood cell).

### Statistical Modeling

An overview of the statistical methods used is summarized in this section; a full discussion is in File S1. For inter- and intra-subject measurements, the coefficient of variation (CV) was defined as standard deviation/mean and was used as a metric for evaluating distributions. MANOVAs were run for eptifibatide, ASA or heparin comparing shear rate (*shear*) or platelet therapy doses (*dose*), controlling for inter-subject variability and analyzing these variables effect on 1/*t_occlusion_*. Using Tukey's posttest all pairwise comparisons between *shear* or *dose* were performed. After pairwise comparisons, Cox proportional hazards (CPH) models were analyzed for each of the therapies to obtain the relative hazard of occlusion as a function of *dose* and *shear*. These models were right censored after 3600 sec. For these CPH models dose and shear were considered continuous variables so that a hazard ratio of occlusion could be used to predict therapeutic efficacies of shear reduction versus therapy administration. Next, a CPH model was constructed in which platelet therapies (*drug*) were directly compared to each other for their efficacy in preventing occlusion. For these estimates antiplatelet therapy efficacy was measured when controlling for shear rate tested and inter- subject variability to ensure no confounding of association between *t_occlusion_* and *drug*. Units of variables, *shear* and *dose*, for all models were chosen based on magnitude of β parameter estimates so that easy comparisons could be made between covariates, the authors recognize that these values are not necessarily clinically relevant but that extrapolation of the estimate can be made across the range of clinically relevant doses tested. All CPH models list a hazard ratio for variables that is constant over the range of values indicated, the cumulative hazard up to any time is then summed to obtain an instantaneous hazard of occlusion.

After comparing the effects of platelet therapies on preventing occlusion, an assessment of the thrombus detachment was performed using a logistic regression. In this logistic model the effect of *drug* on detachment of thrombi (*detach*) (0 = no detachment, 1 = detachment) was measured while controlling for shear rate and therapy dose. Next, the literature was searched and significant predictors of occlusion from CBC parameters were found and used to predict whether eptifibatide had been administered to a patient using a logistic regression (0 = no eptifibatide, 1 = administered eptifibatide). Finally, the sensitivity of *AUC* in predicting platelet therapy delivery was compared to that which had been shown in the 1/*t_occlusion_* MANOVA initially done in this analysis. For all models analyzed in this report an α<0.05 was used for significance. SAS statistical software (SAS Institute Incorporated, Cary, NC) was used to analyze all statistical models.

CPH allows for multivariate survival analysis and provides a powerful potential tool for therapy optimization. In order to use CPH models four assumptions must be made: 1) Covariates have multiplicative effects on the hazard function; 2) Covariates have linear effects on the natural logarithm of the hazard function; 3) Hazard ratio for all subjects is constant over the experiment period; 4) Events are not collinear or tied to each other. The covariates measured (shear, dose, antiplatelet therapy, etc.) do not show additive trends in the data, indicating that occlusion time does not increase by a constant value when at a given antiplatelet therapy level, shear, etc. and thus the first criterion was considered satisfied. A more in-depth discussion of the other assumptions can be found File S1 of this document.

To the knowledge of the authors, 1/*t_occlusion_* has not previously been used as an indicator for occlusion time or efficacy of variables in predicting occlusion events, but has been beneficial to this work due to its ability to be used in general linear models (GLM's) for the analysis of occlusion time. GLM's allow for quantitative comparisons to be made between models using different variables and performed in different conditions by using the R^2^ or adjusted R^2^ metric to quantitatively show the goodness of fit of the model proposed. Currently, no accepted equivalent for R^2^ exists for CPH or logistic models and thus researchers using these models to show efficacy of measured variables or predictive capabilities of treatments cannot directly compare their study to those of other studies in any other way than by showing decreased or increased mortality, number of events, etc. Furthermore, the use of GLMs allows for controlled pairwise comparisons between multiple conditions to be able to be performed without increasing the odds of type 1 error by using such methodologies as Tukey's post-test or a Bonferroni correction for multiple comparisons. Currently, no accepted method allows for this in either CPH or logistic models. This provides further justification for use of 1/*t_occlusion_* in the modeling in this work and furthermore provides a methodology for other researchers in the field to make quantitative comparisons between models.

## Results

**Figure 2:**
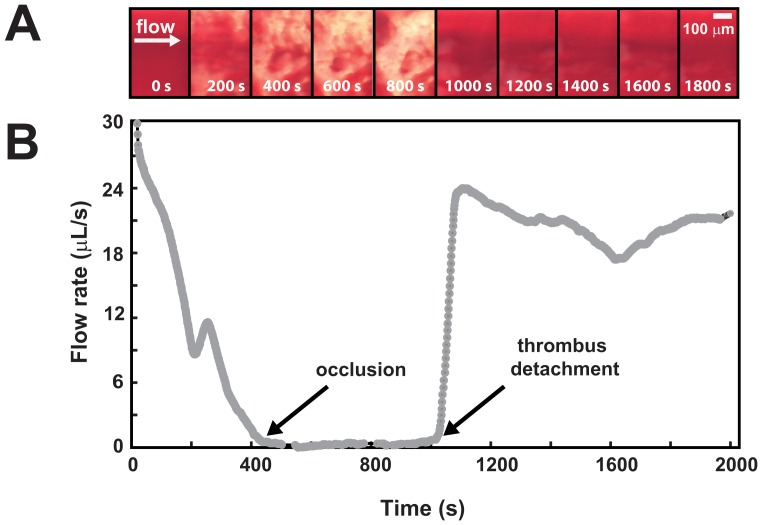
Formation and measurement of thrombus in a channel run within the microfluidic device measured using both microscopy and flow rate at 10000^−1^ initial shear rate. Microscopy images (A) show aggregation initiation at the entry of the stenosis, where brighter areas of the images correspond to more platelet mass. Time stamps for images in seconds are shown at the bottom of each image, and correspond with the time axis shown below in (B). As platelets aggregate, the flow rate (B) decreases until it reaches occlusion time. The unstable thrombus detaches, indicated by the sudden increase in flow rate.

**Figure 3:**
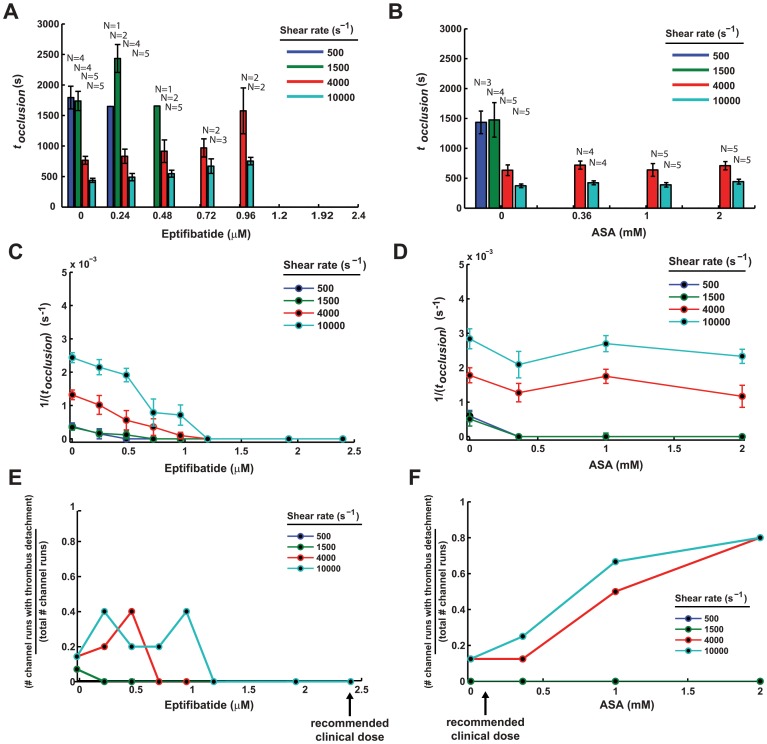
Effects of antiplatelet therapy on occlusion time and thrombus detachment. Occlusion time (*t_occlusion_*) and standard error vs. antiplatelet therapy concentration at a range of initial shear rates for (A) eptifibatide and (B) ASA for N subjects shown. Subjects not shown did not occlude. (C) and (D) represent the inverse of occlusion time and standard error vs. therapy concentration for a range of initial shear rates, corresponding to (A) and (B), respectively. The inverse (1/*t_occlusion_*) includes non-occluded channel runs by assigning them a zero value, thus each point represents pooled data from N = 5 subjects. The likelihood of thrombus detachment, represented as the number of channel runs with thrombus detachment divided by the total number of channel runs is shown vs. therapy concentration for a range of initial shear rates is shown in (E), (F).

Figure 2 shows a typical experimental channel run with the measured occlusion time and a thrombus detachment event. The endpoints of total occlusion by cessation of flow and detachment by sudden increase in flow are matched by images of thrombus in the test section. Figure 3A,B shows occlusion time (*t_occlusion_*) and Figure 3C,D, shows its inverse along with standard error vs. therapy. Although the devices were filled completely with collagen (instead of selectively at the stenosis), we did not observe platelet clotting upstream or downstream of the stenosis region in image recordings or post-test physical dissections of devices during any experiments. We have presented the inverse of occlusion time as more graphically intuitive in that no occlusion tends to zero instead of towards infinity for occlusion time, as described in the statistical methods above. For eptifibatide, occlusion times were lengthened and no occlusion at high doses above 1.2 µM was seen. However, the occlusion time was heavily modulated at high shear rates. Note that occlusion did not occur at any shear rates for the recommended eptifibatide dose of 2.4 µM.

For ASA, occlusion time did not increase at high shear rates of 4000 and 10,000 s^−1^ at both the recommended dose of 0.1 mM up to 2.0 mM. Instead, effects of ASA at recommended low concentrations was seen only at the lower shear rates of 100 and 1500 s^−1^. Figure 3E,F shows likelihood of thrombus detachment, represented as the number of channel runs with thrombus detachment divided by the total number of channel runs for a range of shear rates. For eptifibatide, detachment was eliminated at high doses, where little thrombus was present. For ASA, detachment was more likely at the higher doses, going from about 0.15 to 0.8 as ASA was raised to 2 mM. The statistical significance of these results is detailed below in Section “*Occlusion Times for ASA are Significantly Different Between Shear Rates and Therapy Doses*”. Table 1 lists the continuous variables for occlusion time (*t_occlusion_*), inverse occlusion time (1/*t_occlusion_*) and complete CBC metrics including mean corpuscular volume (*MCV*), red blood cell count (*RBC*), lymphocyte count (*LYM*), mean platelet volume (*MPV*), area under curve (*AUC*), hemoglobin (*HGB*), hematocrit packed cell volume (*HCT*), white blood cell count (*WBC*), and platelet count (*PC*). Table S1 in File S1 contains the categorical variables used for the models subject (*donor*), dose of antiplatelet therapy divided into levels (*dose_cat*), antiplatelet therapy used (*drug*), and shear rate divided into levels (*shear_cat*). Number of measures was defined by number of channel runs which reached occlusion for t_occlusion_, and by all channel runs for the other listed measures.

**Table 1:** Continues variables measured and their relevant statistics.

Variable	No. of Measures	Mean	St. Dev.	Range	Skewness	Kurtosis
***t_occlusion_*** * **(sec)**	139	895.95	606.43	215–2856	1.32	0.94
**1/** ***t_occlusion_*** ** (sec^−1^)**	270	8.2•10^−4^	1.0•10^−4^	0–4.65•10^−3^	0.98	−0.01
***MCV*** ** (fL/cell)**	262	83.98	4.22	74–90.5	−0.80	0.65
***RBC*** ** (Count)**	262	5.04	0.49	4.32–6.21	0.66	−0.12
***LYM*** ** (Count)**	262	1.63	0.39	1.13–2.99	1.92	4.28
***MPV*** ** (fL)**	262	7.26	1.16	5.22–10.8	1.52	3.07
***AUC***	214	47.59	29.27	2.15–108	0.15	−1.28
***HGB*** ** (g/dL)**	262	14.62	0.79	13.3–16.5	0.32	−0.41
***HCT*** ** (%)**	262	42.18	2.38	39–46.6	0.30	−1.15
***WBC*** ** (Count)**	262	5.15	1.00	3.43–7.45	0.46	−0.29
***PC*** ** (Count)**	262	243.32	25.43	179–293	−0.11	−0.09

Indicates that for occlusion time calculations all channel runs that did not occlude were excluded from this analysis, thus the number of measures for 1/*t_occlusion_* is greater than *t_occlusion_*.

### Device Assay was Reproducible Across and Within Subject

To quantify the reproducibility of our assay, we evaluated the mean, standard error, and coefficient of variation of 1/*t*_occlusion_ measurements from T = 5 control trials (saline-treated) for the same N = 1 subject (“intra-subject”), and for T = 1 trials for N = 7 different subjects (“inter-subject”), as shown in Table 2.

**Table 2:** Intra-subject and inter-subject assay variation.

Shear rate (s^−1^)		1/*t*_occlusion_ (•10^−4^ s^−1^)	CV
**500**	Intra-subject	5.1±1.2	0.53
	Inter-subject	4.8±1.2	0.99
**1500**	Intra-subject	4.1±1.4	0.81
	Inter-subject	4.0±1	0.7
**4000**	Intra-subject	16±0.1	0.13
	Inter-subject	16±2.5	0.45
**10000**	Intra-subject	24±2	0.21
	Inter-subject	26±3.2	0.37
**Mean**	Intra-subject		0.42±0.2
	Inter-subject		0.63±0.1

Data for intra-subject metrics is shown for T = 5 (N = 1) trials while inter-subject metrics is shown for N = 7 (T = 1) subjects. Mean 1/*t_occlusion_* are shown plus/minus standard error and coefficient of variation (CV) values. Mean CV values are shown plus/minus standard error.

Both intra-subject and inter-subject CV values were higher at physiological low shear rates (0.67 and 0.85 on average, respectively), and lower at pathological high shear rates (0.17 and 0.41, respectively). Compared to intra-subject variation, inter-subject variation was higher at all shear rates except 1500 s^−1^, and on average the inter-subject CV was 33% greater than intra-subject variation. An interpretation of these variances can be seen in the discussion of this report.

### Occlusion Times for Eptifibatide are Significantly Different Between Shear Rates and Antiplatelet Therapy Doses

The effects of both shear rates and eptifibatide at multiple doses were analyzed. Increasing eptifibatide doses produced increases with *t_occlusion_* in our microfluidic assay at high shear rates, and a loss of occlusion for all shear rates at doses in excess of 1.2 µM, shown in Figure 3A,C. From the GLM it was found that 1/*t_occlusion_* was statistically different for all pairwise comparisons between shear rates, except between the two physiological shear rates 500 and 1500 s^−1^. Eptifibatide dose was also significant, with increases in concentration leading to statistically significant increases in occlusion time between many dose levels. A summary of the significant comparisons can be seen in Table 3. Of note, is that no eptifibatide dose above 0.72 µM was statistically different from higher doses even when controlling for shear rate and inter-subject variability. A full table of all comparisons and their significance can be seen in the Supporting Information Table S2A and S2B in File S1.

**Table 3:** Significance of shear and dose for eptifibatide as judged by MANOVA with Tukey's Posttest.

Variable	Level	Mean 1/*t_occlusion_* (•10^−4^ s^−1^)	Significance Grouping	
	**500**	2.1	A	
			A	
	**1500**	2.2	A	
**shear (s^−1^)**				
	**4000**	8.6	B	
				
	**10000**	17.0	C	
	**0**	13.0		D
				D
	**0.24**	9.1	E	D
			E	
	**0.48**	6.7	E	F
				F
**dose (µM)**	**0.72**	4.0	G	F
			G	
	**0.96**	1.6	G	
			G	
	**1.2**	.	G	
			G	
	**2.4**	.	G	

Means with the same letter for each level of variable are not significantly different as shown in the Significance Grouping Columns.

Next, the hazard ratio and magnitude of importance of shear and eptifibatide dose on occlusion time was determined. The Cox proportional hazards model showed that the hazard of occlusion increased with increasing shear rates and decreased with increasing eptifibatide dose. With each increase in shear of 1000 s^−1^ the hazard of occlusion increased by 1.65 times; while increasing eptifibatide by 0.1 µM decreased the hazard of occlusion by 0.66 times. These hazards were uncovered even when controlling for inter-subject variability. Thus, the effect of shear on creation of occlusive thrombus is shown to be strong and should be considered when selecting the amount of eptifibatide for preventing thrombosis. For example, percutaneous angioplasty or stenting may reduce the local shear rate from 10000 s^−1^ to 4000 s^−1^, which provides a similar reduction in hazard ratio as treatment with 0.72 µM eptifibatide alone (hazard ratio = 0.096 for stenting versus 0.087 for eptifibatide). A table of hazard ratios by variable, confidence intervals, and significance can be seen in the Table S3 in File S1.

### Occlusion Times for ASA are Significantly Different Between Shear Rates and Therapy Doses

The effects of the range of shear rates and ASA antiplatelet therapy were analyzed next in an identical manner to that of eptifibatide. Unlike eptifibatide, all tested doses of ASA were unable to fully prevent occlusive thrombosis at high shear rates 4000 and 10000 s^−1^, even at maximum doses of twenty times (2 mM) the recommended daily oral dose of 100 mg bolus (0.1 mM). In contrast, ASA prevented nearly all occlusive thrombosis at low shear rates 500 and 1500 s^−1^, at even the lowest tested doses as shown in Figure 3B,D. Thus, the ASA may not be effective in preventing arterial thrombosis at high shear rates.

When we examined 1/*t_occlusion_* data using a general linear model, we found that ASA was statistically different for all pairwise comparisons between shear rates except when comparing 500 and 1500 s^−1^ (Table 4). Additionally, ANOVA analysis found that ASA dose increased occlusion time over the parameter space studied. Tukey's post-test identified an effective dose of ASA as any dose of 0.36 µM or higher which were statistically different from lower doses even when controlling for shear rate and inter-subject variability, indicating a saturation of the effect of ASA at this point. A summary of the significant comparisons can be seen in Table 4. A full table of all comparisons and their significance can be seen in the Supporting Information Table S4 in File S1.

**Table 4:** Significance of shear and dose for ASA as judged by MANOVA with Tukey's Posttest.

Variable	Level	Mean 1/*t_occlusion_* (•10^−4^ s^−1^)	Significance Grouping
	500	2.5	A
			A
	1500	2.7	A
**Shear (s^−1^)**			
	4000	15.0	B
			
	10000	22.0	C
	0	13.0	D
			
	0.36	9.8	E
**Dose (µM)**			E
	1.0	12.0	E
			E
	2.0	8.8	E

Means with the same letter for each level of variable are not significantly different as shown in the Significance Grouping Columns.

Next, the hazard ratio and magnitude of importance of shear and ASA dose on occlusion time was determined. The Cox proportional hazards model showed that the hazard of occlusion increased with increasing shear rates and decreased with increasing ASA dose. With each increase in shear of 1000 s^−1^ the hazard of occlusion increased by 1.68 times; while increasing ASA by 1.0 mM decreased the hazard of occlusion by 0.43 times. Notably, the maximum effect of ASA even at twenty times its normal *in-vivo* dose was only able to reduce the hazard of occlusion by 0.17 times compared to an untreated subject. All reductions in pathologic high shear lead to larger decreases in the hazard ratio of occlusion by at least an order of magnitude, except for those from relatively normal shears of 1500 s^−1^ to 500 s^−1^. A full table of all variables hazard ratios, confidence intervals, and significance can be seen in the Supporting Information Table S5 in File S1.

Finally, an identical analysis was done for heparin therapy for doses of 8, 20 or 40 Units/L and results showed identical trends between that of ASA and heparin. Specifically, in Table S6A and S6B in File S1 it was found that there was no statistical difference between any heparin dose above 8 Units/L, indicating a saturation of the effect of heparin after this point. Additionally, all shear rates tested were statistically different except between 500 and 1500 s^−1^. The CPH model showed that the hazard of occlusion of samples treated with heparin increased by 1.68 times per 1000 s^−1^ and decreased by 0.522 times for each unit of heparin delivered.

### Comparisons Between Antiplatelet Therapies Show Significant Differences in Occlusion Time and Thrombus Detachment

In order to directly compare the effects at multiple shear rates of different platelet therapies, and to determine the optimal antiplatelet therapy for specific shear regimes, an additional Cox proportional hazards model was constructed. We found that, occlusion rates were statistically different between the three tested platelet therapies (eptifibatide, ASA and heparin), when controlling for shear rate, dosage and inter-subject variability. When comparing the hazard ratios of ASA and heparin to that of eptifibatide, the analysis found that samples had four times the hazard ratio of occlusion with ASA and nine times with heparin. Furthermore, a sample with no antiplatelet therapy was found to have fourteen times the hazard ratio of occlusion than those treated with eptifibatide. A summary of the comparisons can be seen in Table 5. A full table of detailed statistical measures for this model can be seen in Table S7 in File S1.

**Table 5:** Hazard ratio estimates for three therapies compared to each other.

Parameter	Chi-Square	P-Value	Hazard Ratio	95% Hazard Ratio Confidence Limits	
**shear (per 1000 s^−1^)**	177.467	<.0001	1.476	1.394 -	1.563
**No therapy vs. eptifibatide**	84.3223	<.0001	14.1010	8.016 -	24.806
**ASA vs. eptifibatide**	17.6775	<.0001	4.3090	2.181 -	8.514
**heparin vs. eptifibatide**	8.5027	0.0035	9.0600	2.060 -	39.856
**dose (per µM)**	1.9039	0.1676	1.000	1.000 -	1.000
**donor (1–7)**	<12.3	>0.0005	<7.29	0.318 -	22.1

Next, a logistic regression was created to directly compare the occurrence of thrombus detachment between ASA and eptifibatide. It was found that both ASA and eptifibatide were significant in predicting thrombus detachment. However, an inverse trend was seen for these therapies. The odds ratio between eptifibatide and no therapy treatment was found to be 0.72, indicating lower odds of thrombus detachment if eptifibatide was used than if no therapy was administered. In contrast, ASA had an odds ratio of 4.48, showing that the odds of detachment were almost five times higher when a sample was treated with ASA than if a sample had no therapy administered. Thus, eptifibatide lowered the risk of thrombus detachment while ASA raised it.

Also of note is that the 95% Wald confidence interval for the odds of detachment for eptifibatide versus no therapy encompassed 1 and thus the “true” odds of detachment may not be decreased over that of no therapy treatment. A full table of detailed statistical measures for this model can be seen in S8 in File S1.

### Complete Blood Count Factors and Shear Produce Models that are Predictive of Occlusive Thrombosis and Eptifibatide Administration

In order to determine whether our device had similar utility as compared to the Whole Blood Analyzer's *AUC* metric the odds of eptifibatide treatment was modeled using the significant factors found for prediction of occlusion formation from the studies discussed in the discussion of this report. The logistic regression was found to be capable of predicting antiplatelet therapy as assessed by pseudo R^2^ = 0.68 and HLGF chi-square = 2.8. More interestingly, *AUC* was the only significant factor from the model and that removing it from the model drastically reduced both the pseudo R^2^ and HLGF chi-square probability (data not shown). Table 6 shows the results for this logistic regression.

**Table 6:** Parameter estimates for the prediction of antiplatelet therapy delivery by significant variables from the occlusion formation study.

Parameter	β Estimate	Standard Error	Wald Chi-Square	P-Value	Odds Ratio	95% Wald Confidence Limits	
**intercept**	56.5092	39.5604	2.0404	0.1532	.	.	.
**shear (per 1000 s^−1^)**	0.2081	0.1536	1.8359	0.1754	1.231	0.911 -	1.664
***AUC***	−0.1856	0.0454	16.6895	<.0001	0.831	0.760 -	0.908
***WBC***	0.575	0.7443	0.5969	0.4398	1.777	0.413 -	7.643
***MCV***	0.4703	0.348	1.8264	0.1766	1.6	0.809 -	3.165

**Figure 4:**
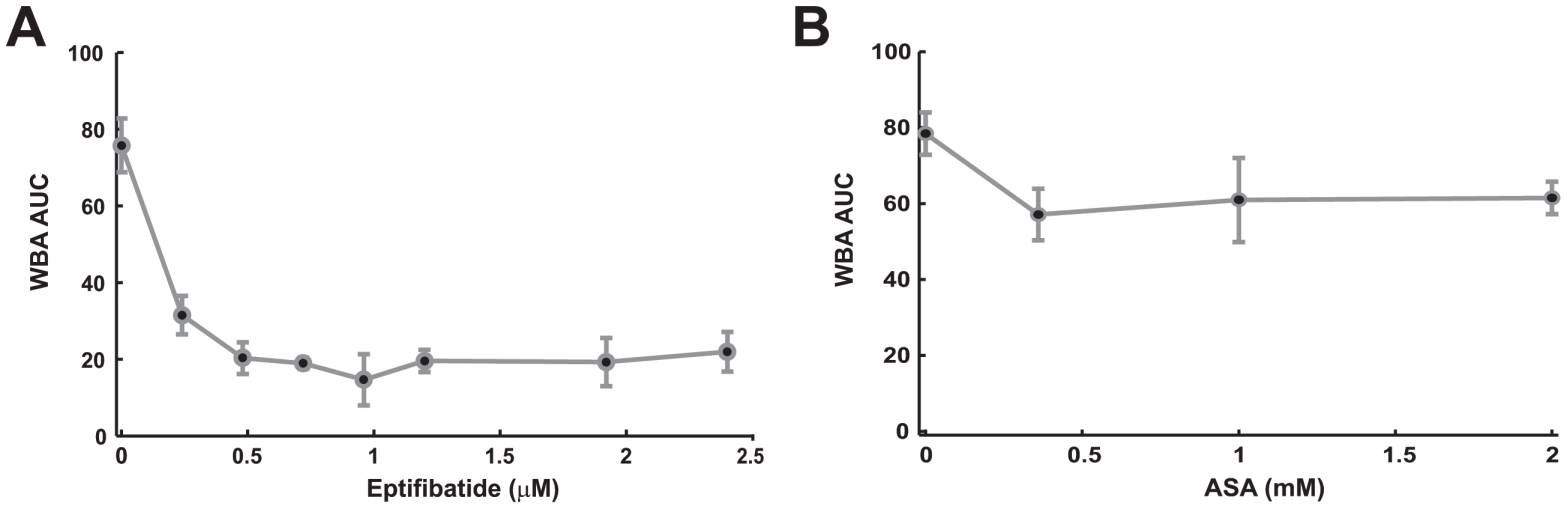
Dose-response measurement using a low shear impedance aggregometer. Plots show results from its Area Under the (Impedance) Curve (AUC) aggregometer metric after the addition of platelet therapies eptifibatide (A) and ASA (B).

To further validate our device against the commercially available WBA low shear aggregometer, the sensitivity of our device to therapy dose was qualitatively compared to that of *AUC* measurements made by WBAs as a function of dose. It was found that WBA showed significant changes in *AUC* after addition of up to 0.72 µg/mL of eptifibatide (Figure 4A) and up to 0.36 mM for ASA Figure 4B). Notably, this range of sensitivity is identical to that shown by our device using 1/*t_occlusion_* metrics shown in Table 3 and Table 4. Table S11A and S11B in File S1 shows the full ANOVA tables for the *AUC* comparisons made by WBA.

## Discussion

The purpose of this study was to provide a systematic, statistical analysis of the simultaneous effects of a wide range of shear rates and doses of aspirin and a GPIIb/IIIa inhibitor (eptifibatide) on occlusive thrombosis and thrombus stability in a microfluidic assay. We examined CBC parameters in our samples prior to testing, measured occlusion times and thrombus detachment, and analyzed our results using statistical models. Additionally, we compared our new method against a commercial WBA platelet analyzer.

Our results showed that shear rate was one of the strongest determinants of occlusion time in any channel run with or without antiplatelet agents. Furthermore, high shear rates had greater effects on increasing hazard ratios than ASA. Direct comparison of the efficacy of ASA vs. eptifibatide showed that the latter was nearly four times more effective at preventing occlusion than the former in our studies. This result was supported by prior clinical findings that eptifibatide provides more potent platelet inhibition in comparison to aspirin and clopidogrel. This is to be expected since eptifibatide directly inhibits the GPIIb/IIIa at high and low shear rates, in contrast to aspirin and clopidogrel which affect GPIIb/IIIa indirectly through the soluble factors and the ADP pathway at lower shear rates [14], [39], [40].

Reproducibility of our assay as measured by the coefficient of variation (CV) was similar to values measured at similar shear rates from other microfluidic studies (intra-subject CV = 0.09±0.3 to 0.55±0.3, inter-subject CV = 0.45±0.2 at 1500 s^−1^ [12] or ∼0.61 at 1200 s^−1^ [41] or ∼0.25 at 1300 s^−1^ [17] or ∼0.61 at 420–32000 s^−1^ [42] or ∼0.17 at 600 s^−1^ [19]). Larger CV's in this study (CV = 0.63±0.1) can also be attributed to increased observation times (here up to 60 min vs. 5 min in [12]) and the measurement of much larger masses of platelets (vs. adhered platelets covering 3–22% of a surface in [12]).

### Antiplatelet Therapy Efficacy in the Prevention of Occlusion Formation

Our results that doses of eptifibatide exceeding 1.2 µM reduces occlusive thrombosis for a range of shear rates support findings by previous groups [17], [43]–[45]. However, new to this report was that hazard ratios from the modeling of occlusion time showed that higher concentrations of eptifibatide are necessary to abrogate the hazard induced by shear increase. Clinically, this has implications for patients with existing cardiovascular pathologies that elevate local shear rates, by indicating that a higher dose of eptifibatide may be needed to counteract the hazard induced by increased shear, although such increased doses should be approached with caution due to potential increased risks for impairment of primary hemostasis and bleeding. Furthermore, that shear rate was significant in all therapies tested indicates that an estimate of this factor for patients will lead to improved prediction of occlusion formation. Thus, the system shows the potential for creating more precise eptifibatide doses, which may reduce the serious bleeding complications associated with GPIIb/IIIa platelet therapies and add a new indication for clinicians to consider when prescribing eptifibatide dose [46], [47].

While statistical analysis showed that general trends in time to occlusion formation were similar between eptifibatide, heparin and ASA across shear and dose, 20× the clinically recommended dose of ASA only reduced the hazard by 0.17 times while 0.72 µM, a third of clinical dose, of eptifibatide reduced the hazard by 0.087 times, almost twice as much. Additionally, lower magnitudes of hazard reduction were found for heparin as compared to ASA. To further support these results the model directly comparing platelet therapies showed that the hazard of occlusion was three times higher for ASA and nine times higher for heparin than with eptifibatide when controlling for dosing of each therapy. Such information could provide guidelines to aid clinicians in the selection of antiplatelet therapy dose—for titrating doses of aspirin to effective concentrations for in some subjects, and/or for the prevention of eptifibatide overdose to reduce bleeding risk. Although it is possible that the combined use of ASA with low concentrations of heparin may mask some of the fibrin-inhibiting effects of the former alone on preventing thrombus formation at arterial shear rates, the formation of stable thrombi at arterial shear rates in both ASA and eptifibatide treated samples implies that these effects do not heavily impact this study.

In contrast to eptifibatide, ASA uses a different mechanism of action by directly inhibiting the enzymes cyclooxygenase-1 and -2 (COX-1, COX-2) from acting on arachidonic acid to produce the soluble factor TXA2 from platelets which enhances aggregation [48]. Thus, one common definition of “aspirin resistance” for commercial tests is the measurement of platelet activity after the addition of arachidonic acid, with “resistant” patients retaining the ability to aggregate platelets [13]. However, the relationship between the initial conversion of arachidonic acid through a number of subsequent downstream events whose activity may be closely linked to fluid transport and ultimate clinical outcomes (e.g. mortality, myocardial or cerebral infarction) is unclear [49]. Such testing procedures often rely on saturating initial concentrations of arachidonic acid within enclosed, stirred volumes instead of single-pass dynamic flow systems, which further reduces physiological relevance [1]. To test whether our ASA protocol was effective at eliminating COX activity, we conducted parallel tests with the whole blood analyzer using arachidonic acid instead of collagen as our agonist, and found that thrombosis was subsequently inhibited (data not shown). Thus, although our ASA testing procedure was successfully able to effectively eliminate COX-1 activity, it was still unable to eliminate thrombosis at all shear rates even at 20× recommended doses.

Our study showed little change in occlusion times, but large increases in thrombus detachment after addition of ASA. One potential reason for these results may be ASA's reduced efficacy when added *in-vitro* as opposed to *in-vivo*, a topic which has been explored by recent studies [50]. Despite such potential alterations, results of this study are supported by previous *ex-vivo* and *in-vivo* clinical studies. For example, one *ex-vivo* study of blood samples from patients who had orally ingested and metabolized ASA reported that thrombosis is unaffected by ASA in eccentric stenoses with shear rates of 10500 s^−1^ [51], while at the lower shear rate of 2600 s^−1^ the size of formed thrombus and fibrin deposition were significantly reduced [52]. In contrast, at low shear rates of 2600 s^−1^, platelet binding has been found to be more dependent on shape change, fibrin deposition, and fibrinogen bonds—factors known to be affected by soluble agonists such as ASA [40]. This may be a result of multiple factors. First, ASA loses efficacy when shear enhances transport of soluble factors such as TXA2 and COX-1 (primary targets of ASA) away from the growing thrombus. Additionally, platelet shape changes induced by soluble factors is not necessary for the formation of thrombi at high shear rates [53]. Enhanced thrombus detachment after ASA treatment noted in this study has also been recorded by previous authors at physiological shear rates of 1500 s^−1^ [54]. Our results are also supported by *in-vivo* clinical trials which have reported “no antithrombotic benefit…in patients with severe atherosclerosis and stenoses…however anti-thrombotic protection…in patients with less severe lesions is reported” and that high dose ASA therapy was ineffective at preventing re-occlusion in high shear stenoses (>90%) [40], [55]. Previous groups have also used microfluidic flow systems to show similarly low efficacy of ASA at arterial shear rates (1500 s^−1^) in comparison to other GPIIb/IIIa inhibitors such as abciximab [26] or P_2_Y_12_ inhibitors such as MeSAMPs [56]. Thus, our results show good agreement with a variety of other *in-vivo* and *ex-vivo* studies on the effects of ASA on thrombus detachment and on the relative magnitude of ASA in comparison to alternative therapies including GPIIb/IIIa inhibitors.

### Modeling of CBC Data with Shear Implicates its Importance for Prediction of Occlusion Formation

Factors that were analyzed in our model were *AUC*, *WBC* and *MCV*. *MCV* was chosen because increased cell number and size leads to occlusion due to platelet margination [57]. Additionally, increased numbers of WBC's and inflammation have been associated with increased myocardial infarct events [58]. Finally, *AUC* was chosen because the goal of the model was to determine whether our device had similar utility as compared to the Whole Blood Analyzer's *AUC* metric in prediction of therapy administration. Using our device and modeling the AUC with several other factors obtained from CBC counts it was found that our device was able to recapitulate the predicative ability of the AUC metric.

Comparison with the WBA shows a range of sensitivity for addition of eptifibatide identical to that shown by our device using 1/*t_occlusion_* metrics. Notably, the WBA has previously been successfully used for the detection of clinically effective doses of eptifibatide from venous blood samples nearly 72 hours after its injection into subjects undergoing coronary stenting [59]. Thus, our system should be able to achieve similar levels of clinical utility when testing samples at physiological shear rates while adding clinically relevant additional high shear rate information and thrombus detachment metrics, all factors which this work has shown may inform improved patient dosing.

## Conclusions

This paper presents a microfluidic study of the effects of a spectrum of whole blood shear rates and antiplatelet therapies on occlusive thrombosis and thrombus detachment. We have shown that antiplatelet efficacy has significant effects at high pathological shear rates compared to normal physiologic shear rates. The effectiveness of ASA against high shear thrombosis was several orders of magnitude lower than that of eptifibatide, even at doses 20 times clinically recommended concentrations. Notably, high shear thrombi formed with ASA were found to be more likely to detach than those formed without ASA, or with eptifibatide. Eptifibatide reduced the occurrence of occlusion when controlling for shear rate, and its efficacy increased at all shear rates with dose concentration.

There are two potential clinical implications of these findings. First, ASA is widely prescribed to reduce the risk of myocardial infarct and stroke, although resistance to such treatment is common and its effectiveness has been found to vary over time. Our finding that high shear thrombi are not uniformly prevented by ASA may explain the phenomenon of “aspirin resistance” as a local flow mechanics factor independent of platelet activation. Second, ASA may contribute to unstable thrombi detachment with the shedding of larger emboli as a different mechanism for clinical failure with ASA. For both thrombus formation and detachment, local high shear rates are important. Thus, future clinical tests characterizing causes for aspirin resistance may need to include high shear rates from angiographic images as indicators of arterial thrombus in addition to genetic testing and platelet reactivity data.

While this microfluidic device affords low blood volumes and high throughput, its reproduction of in-vivo conditions is limited. The steady flow through rectangular channels do not produce pulsatile secondary flow patterns in arteries [60]. The assay requires more sample volume than others, and is thus not currently suitable for point-of-care applications. Clinical studies should be performed to relate occlusive thrombus accumulation in the device to long-term clinical outcomes. This work represents a quantitative comparison of the effects of high shear rate and antiplatelet therapy dose on thrombosis.

Effects of shear rate and dose parameters were analyzed using general linear models, logistic regressions, and Cox proportional hazards models—techniques often used in multi-parameter clinical studies. We showed the system's ability to evaluate antiplatelet therapy with sensitivity comparable to whole blood aggregometry. By providing quantitative dose-response curves of the effects of antiplatelet therapy under a wide range of shear rates, this study furthers understanding of thrombosis and antiplatelet therapy resistance, which is expected to be utilized in the construction of more accurate models for optimizing treatment.

## Supporting Information

File S1 This file contains extended descriptions, equations, and results for the statistical modeling methods employed in this work. Additionally this file contains Tables S1–S11.
(DOC)
