# Supplementary material for: Microfluidic Thrombosis under Multiple Shear Rates and Antiplatelet Therapy Doses
Source: PLoS One. 2014 Jan 3;9(1):e82493. doi: 10.1371/journal.pone.0082493 (PMC3880267; doi:10.1371/journal.pone.0082493)
Supplement: File S1 — This file contains extended descriptions, equations, and results for the statistical modeling methods employed in this work. Additionally this file contains Tables S1–S11. (DOC) [file pone.0082493.s001.doc]

## **Supporting Information**

## Statistical Modeling

### Variable Analysis and Suitability for Modeling

The 1/*t_occlusion_* time had a Skewness of 0.98 and a Kurtosis of -0.01, which are both associated with only mild non-normality (See Table 1). Furthermore, homoscedasticity, or homogeneity of the variance, of the data has been historically shown for occlusion times by many groups and thus was seen as validated [12]. In all models collinearity of variables was assessed and no measured variables were found to be collinear and measurements were independent. Thus, assumptions for linear and logistic regressions were met. To validate that covariates had linear effects on the natural log of the hazard function for the CPH models, models were run omitting each covariate variable of interest and calculating the Martingale residual of that model. A plot of the Martindale residuals versus the analyzed variable was then made and it was found that all residuals formed a straight line (data not shown). This indicates the linear effect of each covariate on the natural log of the hazard.

The hazard ratio for each subject was identical over time as experimental conditions did not change throughout all testing, thus every subject had an equal chance of occlusion throughout the course of all experiments. No variables were found to vary collinearly and all were chosen to be independent variables and thus were not tied to each other. Linear and logistic regression models use fewer assumptions than do CPH models. Normality of 1/*t_occlusion_* was shown and all covariates in every model were independently measured and were not collinear. Also, the pseudo R^2^ was measured for each logistic regression and always found to be above 0.7 and thus that the conditional probabilities were a logistic function of the covariates was seen as validated.

### Analysis of the Effects of Shear and Dose on Occlusion Time

Model 1 shows the general linear model that has 1/*t_occlusion_* as an outcome variable and is a function of shear rate, antiplatelet therapy dose, and subject all modeled as the categorical variables shown in Table S1. For occlusion times longer than 3600 seconds 1/*t_occlusion_* was assumed to be 0. This implies that if occlusion had not occurred within 3600 seconds of a channel run, it would not form at any time afterwards. This assumption is not always true, however, and while this assumption does introduce error into the model it has several strong advantages that are discussed at length in the discussion (section 5.2) of this report. Furthermore, models using an increasingly smaller integer in place of 0 for long occlusion times, showed that any occlusion time longer than ~3500 seconds produced no meaningful changes in the significance of variables or in β coefficients calculated for measured variables.

Model 1 sought to isolate and compare the effects of shear rate or antiplatelet therapy dose when controlling for subject variability. The null hypotheses tested by this model were that 1) no shear rate would be statistically different (as measured by T values and Tukey’s Post Test) from any other shear rate when controlling for antiplatelet therapy dose and inter-subject variability, and 2) that no antiplatelet therapy dose would be statistically different (as measured by T values and Tukey’s Post Test) from any other antiplatelet therapy dose when controlling for shear rate used and inter-subject variability. A reference shear rate and antiplatelet therapy dose were chosen as 10000 s^-1^ and zero concentration, respectively, to reflect a typical subject with atherosclerosis who would require optimization of antiplatelet therapy. Finally, the variable *donor* was included in the analysis to account for the repeated measures of each subject across shears and doses and to help limit the large inter-subject variability that is seen with primary subjects. The reference subject was chosen at random and because all other factors control for subject in their calculation of beta, and no influence on the calculated coefficients or their significance was observed when changing between reference subjects. Thus, the reference group was any subject run at a 10000 s^-1^ shear rate with no antiplatelet therapy administered. Model 1 was used to show the effect of *shear_cat* and *dose_cat* on three different platelet therapies: eptifibatide, ASA, and heparin. An identical model was used for each antiplatelet therapy. A MANOVA with Tukey’s Posttest was used to identify the significance of all pairwise comparisons variables with an α <0.05 used for significance.

| 1/*t_occlusion_* = β_1_ + β_2_**shear_cat* + β_3_**dose_cat* + β_4_**donor* | **Model 1** |
| --- | --- |
|  |  |

### Assessment of the Magnitude of Importance of Shear and Antiplatelet Therapy Dose on Occlusion Times for Three Platelet Therapies

Model 1 showed that different shear rates and doses of antiplatelet therapy administered were statistically different from each other. Next, the magnitude of the effect of dose or shear for each therapy was evaluated for the hazard of occlusion at different levels of these factors. Cox proportional hazards models were created in which the occlusion time was modeled as a function of shear rate, dose of antiplatelet therapy and subject. The null hypothesis for this model was that *shear* and *dose* would not be significant factors in prediction of occlusion time. Additionally, an assessment of the magnitude of influence and hazard ratio for each of these factors had relative to each other was of interest in these models. Shear rate and dose were continuous variables where shear was modeled by increments of 1000 s^-1^ and dose of antiplatelet therapy was modeled in increments of 100 nM for eptifibatide, 1.0 mM for ASA and Units/L for heparin. Right censoring for these models was performed at 3600 seconds. Model 2 shows the model used for each of the three platelet therapies. For each antiplatelet therapy the reference subject was chosen at random and because all other factors control for subject in their calculation of beta no influence on the calculated coefficients or their significance was seen when changing between reference subjects. The hazard coefficients of the donors themselves represent the relative hazard of occlusion of a subject compared to a reference subject. Because the reference subject was chosen at random all interpretation of hazard of occlusion for donors is relative and thus no real meaning can be drawn from these coefficients. *Donor* was kept in the model to account for the repeated measures from subjects and to help account for the large inter-subject variability seen in primary donors. Chi-square tests were performed to identify the significance of a variable with an α <0.05 used for significance.

| log H(*t_Occlusion_)* = β_1_ + β_2_**shear* + β_3_**dose* + β_4_**donor* | **Model 2** |
| --- | --- |
|  |  |

### Comparative Assessment of Three Different Antiplatelet Therapies at Preventing Occlusion

Model 1 and Model 2 showed the importance of shear rate and antiplatelet therapy dose on occlusion times however, these models were not able to evaluate the relative efficacy of each antiplatelet therapy in preventing occlusion. Thus, a Cox proportional hazards model was created in which the occlusion time was modeled as a function of shear rate, therapy treatment, and subject. The null hypothesis tested by Model 3 was that there would be no significant difference in occlusion time between any therapy and eptifibatide. For these models shear rate and therapy dose were continuous variables where shear was modeled as per 1000 s^-1^ and *dose* was modeled as per 1 µM/ In Model 3 *drug* and *donor* were categorical variables. Right censoring for these models was performed at 3600 seconds. Model 3 shows the model used. For this model the reference subject was chosen at random and because all other factors control for subject in their calculation of beta no influence on the calculated coefficients or their significance was seen when changing between reference subjects. The hazard coefficients of the donors themselves represent the relative hazard of occlusion of a subject compared to a reference subject. Because the reference subject was chosen at random all interpretation of hazard of occlusion for donors is relative and thus no real meaning can be drawn from these coefficients. *Donor* was kept in the model to account for the repeated measures from subjects and to help account for the large inter-subject variability seen in primary donors. Thus, for Model 3 the reference group was any subject run at a 0 s^-1^ shear rate with eptifibatide administered. Eptifibatide was chosen as the reference group for Model 3 because the hazard ratio between each of the other therapies tested and eptifibatide would enable direct comparison of the relative hazard of occlusive thrombosis using each type of antiplatelet therapy. Chi-square tests were performed to identify the significance of a variable with an α <0.05 used for significance.

| log H(t*_occlusion_)* = β_1_ + β_2_**shear* + β_3_**drug* + β_4_**dose* + β_5_**donor* | **Model 3** |
| --- | --- |

### Assessment of the Effect of Antiplatelet Therapy on Thrombus Detachment

Model 3 showed significant differences in thrombus detachment between the ASA and eptifibatide. However, whether the device was able to distinguish between these modes of actions was desired. Thus, a logistic regression was created to predict an outcome other than occlusion time, thrombus detachment. Thrombus detachment was modeled as a binary event, either thrombi detached during the measured period (3600 seconds) or they did not. Model 4 shows detachment events as a function of *shear*, *drug*, and *donor*. The null hypothesis tested by Model 4 was that there would be no significant difference in detachment events between eptifibatide and ASA. For these models *shear* was a continuous variable where shear was modeled as per 1000 s^-1^ and *drug* and *donor* were categorical variables. Heparin was not included in this model due to its collinearity with ASA. The reference subject for Model 4 was chosen at random and because all other factors control for subject in their calculation of beta no influence on the calculated coefficients or their significance was seen when changing between reference subjects. Thus, for Model 4 the reference group was any subject run at 0 s^-1^ shear rate with ASA administered. Eptifibatide was chosen as the reference group for Model 4 because it is known to have a different mode of prevention from that of ASA and thus the relative risk ratio from using Eptifibatide over that of ASA desired. Wald Chi-square tests were performed to identify the significance of a variable with an α <0.05 used for significance.

| Logit (*detach)* = β_1_ + β_2_**shear* + β_3_**drug* + β_3_**dose* | **Model 4** |  |
| --- | --- | --- |
|  |  |  |
|  |  | |

### Ability of Device to Discern Antiplatelet Therapy Delivery.

A logistic regression was created where all significant variables from **Model 5** were tested for their ability to predict eptifibatide administration. Thus, the delivery of eptifibatide was modeled as a function of *shear*, *AUC*, *WBC*, *RBC*, and *MCV*. Antiplatelet therapy dose was not included in this model because Model 5 was trying to determine if a antiplatelet therapy was being delivered and thus the dose of the therapy would necessarily be tied with therapy delivery. The null hypothesis tested by Model 5 was that *AUC* would not be a significant predictor of Eptifibatide administration. In this model all CBC variables and *shear* were continuous variables. Wald Chi-square tests were performed to identify the significance of a variable and pseudo R^2^ values and HLGF statistics were used to discern the models ability to predict antiplatelet therapy.

| Logit (*eptifibatide)* = β_1_ + β_2_**shear* + β_3_**AUC*+ β_4_**WBC* + β_5_**MCV* | **Model 5** |
| --- | --- |
|  |  |

Finally, a model comparing the sensitivity of AUC versus antiplatelet therapy dose was created so that a quantitative comparison between AUC and the results from Model 1 was performed. Tukey’s posttest was used for all pairwise comparisons between eptifibatide and ASA doses in predicting AUC. An α< 0.05 was used for significance.

### Linear and Cox Proportional Hazards Model Prediction of Shear and Antiplatelet Therapy Dose

Table S1: Frequency and percent of the number of repeated measures of each of the categorical variables measured.

| **Variable** | **Values** | **# of Repeats Measured** | **% of Total** |
| --- | --- | --- | --- |
| ***Subject or Donor*** |  |  |  |
|  | 1 | 24 | 8.9 |
|  | 2 | 74 | 27.4 |
|  | 3 | 30 | 11.1 |
|  | 4 | 16 | 5.9 |
|  | 5 | 16 | 5.9 |
|  | 6 | 16 | 5.9 |
|  | 7 | 66 | 24.4 |
|  | 8 | 28 | 10.4 |
| ***drug*** |  |  |  |
| **None** | 0 | 77 | 28.5 |
|  | 0.24 | 19 | 7.0 |
|  | 0.48 | 20 | 7.4 |
| **Eptifibatide (µM)** | 0.72 | 20 | 7.4 |
| **(T= 109, 28.5%)** | 0.96 | 20 | 7.4 |
|  | 1.20 | 20 | 7.4 |
|  | 2.40 | 20 | 3.7 |
|  | 0.36 | 20 | 7.4 |
| **ASA (mM)** | 1.00 | 24 | 8.9 |
| **(T= 64, 23.7%)** | 2.00 | 20 | 7.4 |
|  | 8 | 4 | 1.5 |
| **heparin (Units/L)** | 20 | 12 | 4.4 |
| **(T= 20, 7.40%)** | 40 | 4 | 1.5 |
| ***shear* (s^-1^)** |  |  |  |
|  | 500 | 63 | 23.3 |
|  | 1500 | 69 | 25.6 |
|  | 4000 | 69 | 25.6 |
|  | 10000 | 69 | 25.6 |

Table S2: ANOVA tables for shear rate and eptifibatide dose showing all pairwise comparisons’ significance as measured by Tukey’s Posttest.

**A)**

| **Least Squares Means for Effect of *Shear*** | | | | |
| --- | --- | --- | --- | --- |
| **Shear (s^-1^)** | **500** | **1500** | **4000** | **10000** |
| **500** |  | 0.9999 | <.0001 | <.0001 |
| **1500** | 0.9999 |  | <.0001 | <.0001 |
| **4000** | <.0001 | <.0001 |  | <.0001 |
| **10000** | <.0001 | <.0001 | <.0001 |  |

**B)**

| **Least Squares Means for Effect of Eptifibatide Dose** | | | | | | | |
| --- | --- | --- | --- | --- | --- | --- | --- |
| **Dose (µM)** | **0** | **0.24** | **0.48** | **0.72** | **0.96** | **1.2** | **2.4** |
| **0** | . | 0.7202 | 0.0382 | <.0001 | <.0001 | <.0001 | <.0001 |
| **0.24** | 0.7202 | . | 0.8809 | 0.0756 | 0.0006 | <.0001 | <.0001 |
| **0.48** | 0.0382 | 0.8809 | . | 0.6697 | 0.0375 | 0.0015 | 0.0006 |
| **0.72** | <.0001 | 0.0756 | 0.6697 | . | 0.7643 | 0.1947 | 0.0522 |
| **0.96** | <.0001 | 0.0006 | 0.0375 | 0.7643 | . | 0.962 | 0.554 |
| **1.2** | <.0001 | <.0001 | 0.0015 | 0.1947 | 0.962 | . | 0.9474 |
| **2.4** | <.0001 | <.0001 | 0.0006 | 0.0522 | 0.554 | 0.9474 | . |

Table S3: Full statistics for CPH model of occlusion time versus eptifibatide *dose*, *shear*, and *donor*.

| **Analysis of Maximum Likelihood Estimates** | | | | | | | | |
| --- | --- | --- | --- | --- | --- | --- | --- | --- |
| **Parameter** |  | **β**  **Estimate** | **Standard Error** | **Chi-Square** | **P-Value** | **Hazard Ratio** | **95% Hazard Ratio Confidence Limits** | |
| ***donor*** | 1 | -0.5370 | 0.5818 | 0.8518 | 0.3560 | 0.584 | 0.187 - | 1.828 |
| ***donor*** | 2 | 0.8005 | 0.4568 | 3.0709 | 0.0797 | 2.227 | 0.910 - | 5.451 |
| ***donor*** | 3 | -0.3193 | 0.5149 | 0.3846 | 0.5351 | 0.727 | 0.265 - | 1.993 |
| ***donor*** | 4 | 2.4238 | 0.6774 | 12.801 | 0.0003 | 11.28 | 2.992 - | 42.58 |
| ***donor*** | 5 | 1.4981 | 0.6670 | 5.0453 | 0.0247 | 4.473 | 1.210 - | 16.53 |
| ***donor*** | 6 | 1.1303 | 0.6657 | 2.8825 | 0.0895 | 3.096 | 0.840 - | 11.41 |
| ***donor*** | 7 | 1.3706 | 0.4661 | 8.6472 | 0.0033 | 3.938 | 1.579 - | 9.817 |
| ***shear*** | per 1000 s^-1^ | 0.4998 | 0.0429 | 135.57 | <.0001 | 1.648 | 1.515 - | 1.793 |
| ***dose*** | per 0.1 µM | -0.0410 | 0.0425 | 93.333 | <.0001 | 0.663 | 0.610 - | 0.721 |

Table S4: ANOVA tables for shear rate and ASA dose showing all pairwise comparisons’ significance as measured by Tukey’s Posttest.

| **Least Squares Means for Effect of Shear** | | | | |
| --- | --- | --- | --- | --- |
| **Shear (s^-1^)** | **500** | **1500** | **4000** | **10000** |
| **500** |  | 0.9993 | <.0001 | <.0001 |
| **1500** | 0.9993 |  | <.0001 | <.0001 |
| **4000** | <.0001 | <.0001 |  | <.0001 |
| **10000** | <.0001 | <.0001 | <.0001 |  |
|  |  |  |  |  |
| **Least Squares Means for Effect of ASA Dose** | | | | |
| **Dose (µM)** | **0** | **0.36** | **1** | **2** |
| **0** |  | 0.0001 | 0.023 | <.0001 |
| **0.36** | 0.0001 |  | 0.391 | 0.8864 |
| **1** | 0.0232 | 0.3906 |  | 0.094 |
| **2** | <.0001 | 0.8864 | 0.094 |  |

Table S5: Full statistics for CPH model of occlusion time versus ASA *dose*, *shear*, and *donor*.

| **Analysis of Maximum Likelihood Estimates** | | | | | | | | |
| --- | --- | --- | --- | --- | --- | --- | --- | --- |
| **Parameter** |  | **β**  **Estimate** | **Standard Error** | **Chi-Square** | **P-Value** | **Hazard Ratio** | **95% Hazard Ratio Confidence Limits** | |
| ***donor*** | 1 | 0.15729 | 0.91803 | 0.0294 | 0.864 | 1.17 | 0.194 - | 7.075 |
| ***donor*** | 2 | 0.7967 | 0.73751 | 1.167 | 0.28 | 2.218 | 0.523 - | 9.414 |
| ***donor*** | 3 | -0.49801 | 0.83163 | 0.3586 | 0.5493 | 0.608 | 0.119 - | 3.102 |
| ***donor*** | 4 | 1.92885 | 0.79968 | 5.8179 | 0.0159 | 6.882 | 1.436 - | 32.989 |
| ***donor*** | 5 | 1.01131 | 0.78668 | 1.6526 | 0.1986 | 2.749 | 0.588 - | 12.848 |
| ***donor*** | 6 | 1.50842 | 0.78828 | 3.6617 | 0.0557 | 4.52 | 0.964 - | 21.188 |
| ***donor*** | 7 | 1.34283 | 0.74402 | 3.2574 | 0.0711 | 3.83 | 0.891 - | 16.463 |
| ***shear*** | Per 1000 s^-1^ | 0.51921 | 0.04633 | 125.5982 | <.0001 | 1.681 | 1.535 - | 1.84 |
| ***dose*** | Per 1.0 mM | -0.84388 | 0.20297 | 17.2857 | <.0001 | 0.43 | 0.289 - | 0.64 |

Table S6: (A) ANOVA tables for shear rate and heparin dose showing all pairwise comparisons’ significance as measured by Tukey’s Posttest. (B) Full statistics for CPH model of occlusion time versus ASA *dose*, *shear*, and *donor*.

**A**

**B**

| **Least Squares Means for Effect of Shear** | | | | |
| --- | --- | --- | --- | --- |
| **Shear (s^-1^)** | **500** | **1500** | **4000** | **10000** |
| **500** |  | 0.9918 | <.0001 | <.0001 |
| **1500** | 0.9918 |  | <.0001 | <.0001 |
| **4000** | <.0001 | <.0001 |  | <.0001 |
| **10000** | <.0001 | <.0001 | <.0001 |  |
|  |  |  |  |  |
| **Least Squares Means for Effect of Heparin Dose** | | | | |
| **Dose (Units/L)** | **3.5** | **8** | **20** | **40** |
| **3.5** |  | 0.3696 | 0.0009 | 0.004 |
| **8** | 0.3696 |  | 0.8921 | 0.5167 |
| **20** | 0.0009 | 0.8921 |  | 0.7288 |
| **40** | 0.004 | 0.5167 | 0.7288 |  |

### Interaction of Platelet Therapies to Predict Occlusion and Thrombus Detachment

Table S7: Complete statistics comparative hazards of three platelet therapies

| **Analysis of Maximum Likelihood Estimates** | | | | | | | | |
| --- | --- | --- | --- | --- | --- | --- | --- | --- |
| **Parameter** |  | **β**  **Estimate** | **Standard Error** | **Chi-Square** | **P-Value** | **Hazard Ratio** | **95% Hazard Ratio Confidence Limits** | |
| ***donor*** | 1 | -0.01411 | 0.57793 | 0.0006 | 0.9805 | 0.986 | 0.318 - | 3.061 |
| ***donor*** | 2 | 0.85205 | 0.45287 | 3.5398 | 0.0599 | 2.344 | 0.965 - | 5.695 |
| ***donor*** | 3 | 0.21462 | 0.50592 | 0.18 | 0.6714 | 1.239 | 0.460 - | 3.341 |
| ***donor*** | 4 | 1.98666 | 0.56677 | 12.2865 | 0.0005 | 7.291 | 2.401 - | 22.143 |
| ***donor*** | 5 | 1.31458 | 0.5553 | 5.6043 | 0.0179 | 3.723 | 1.254 - | 11.056 |
| ***donor*** | 6 | 1.61364 | 0.55005 | 8.6061 | 0.0034 | 5.021 | 1.708 - | 14.757 |
| ***donor*** | 7 | 1.33279 | 0.45142 | 8.7171 | 0.0032 | 3.792 | 1.565 - | 9.185 |
| ***shear* (per 1000 s^-1^)** | | 3.96E-04 | 2.98E-05 | 175.8029 | <.0001 | 1 | 1.000 - | 1.000 |
| **No antiplatelet therapy vs. eptifibatide** | | 2.64626 | 0.28818 | 84.3223 | <.0001 | 14.101 | 8.016 - | 24.806 |
| **ASA vs. eptifibatide** | | 1.46076 | 0.34743 | 17.6775 | <.0001 | 4.309 | 2.181 - | 8.514 |
| **heparin vs. eptifibatide** | | 2.20391 | 0.75581 | 8.5027 | 0.0035 | 9.06 | 2.060 - | 39.856 |
| ***Dose* (per 1 µM)** | | -3.8E-05 | 2.79E-05 | 1.9039 | 0.1676 | 1 | 1.000 - | 1.000 |

Table S8: Complete statistics for analysis of odds ratios of occlusion detachment.

| **Analysis of Maximum Likelihood Estimates** | | | | | | | |
| --- | --- | --- | --- | --- | --- | --- | --- |
| **Parameter** | **Estimate** | **Standard Error** | **Wald Chi-Square** | **P- Value** | **Odds Ratio** | **95% Wald Confidence Limits** | |
| **Intercept** | -5.3898 | 54.8767 | 0.0096 | 0.9218 | . | . | . |
| **500 vs. 10000** | -9.7442 | 164.6 | 0.0035 | 0.9528 | <0.001 | <0.001 - | >999.999 |
| **1500 vs. 10000** | 1.0213 | 54.8807 | 0.0003 | 0.9852 | 0.032 | 0.004 - | 0.261 |
| **4000 vs. 10000** | 4.269 | 54.8765 | 0.0061 | 0.938 | 0.831 | 0.357 - | 1.933 |
| ***dose* (per 1 µM)** | 0.0786 | 0.389 | 0.0408 | 0.8398 | 1.082 | 0.505 - | 2.319 |
| **eptifibatide vs no therapy** | -0.7236 | 0.313 | 5.3428 | 0.0208 | 0.715 | 0.202 - | 2.524 |
| **ASA vs no therapy** | 1.111 | 0.3369 | 10.8743 | 0.001 | 4.475 | 1.182 - | 16.937 |

### CBC Comparison to Shear and Dose for Application to Clinical Systems

Table S9: Optimized logistic model parameter estimates for odds ratio and relative importance of shear and antiplatelet therapy dose on occurrence of occlusion when modeling patients who have received eptifibatide.

| **Parameter** | **β**  **Estimate** | **Standard Error** | **Wald Chi-Square** | **P-Value** | **Odds Ratio** | **95% Wald Confidence Limits** | |
| --- | --- | --- | --- | --- | --- | --- | --- |
| **Intercept** | -53.2198 | 18.9981 | 7.8474 | 0.0051 | . | . | . |
| ***shear***  **(1000 s^-1^)** | 0.6962 | 0.1396 | 24.8733 | <.0001 | 2.006 | 1.526 - | 2.637 |
| ***dose* (per 1 µM)** | -8.5254 | 1.7525 | 23.6647 | <.0001 | <0.001 | <0.001 - | 0.006 |
| ***AUC*** | -0.0303 | 0.0155 | 3.8125 | 0.0500 | 0.97 | 0.941 - | 1.00 |
| ***WBC*** | -1.2275 | 0.3988 | 9.4711 | 0.0021 | 0.293 | 0.134 - | 0.64 |
| ***MCV*** | 0.556 | 0.1673 | 11.0454 | 0.0009 | 1.744 | 1.256 - | 2.42 |

Table S10: Optimized logistic model parameter estimates for odds ratio and relative importance of shear and antiplatelet therapy dose on occurrence of occlusion when modeling patients who have not received eptifibatide.

| **Variable** | **β Estimate** | **Standard Error** | **Wald Chi-Square** | **P-Value** | **Odds Ratio** | **95% Wald Confidence Interval** | |
| --- | --- | --- | --- | --- | --- | --- | --- |
| **intercept** | 5.1444 | 2.2575 | 5.193 | 0.0227 | . | . | . |
| ***shear***  **(per 1000 s^-1^)** | 0.6334 | 0.2743 | 5.3305 | 0.021 | 1.884 | 1.10 - | 3.225 |
| ***WBC*** | -0.9644 | 0.4208 | 5.252 | 0.0219 | 0.381 | 0.167 - | 0.870 |

Table S11: ANOVA tables for shear rate and eptifibatide dose showing all pairwise comparisons’ significance as measured by Tukey’s Posttest.

**A)**

| **Least Squares Means for Effect of ASA Dose** | | | | |
| --- | --- | --- | --- | --- |
| **Dose (µM)** | **0** | **0.36** | **1** | **2** |
| **0** |  | 0.0192 | 0.9795 | 0.9518 |
| **0.36** | 0.0192 |  | 0.401 | 0.2092 |
| **1** | 0.9795 | 0.401 |  | 0.9033 |
| **2** | 0.9518 | 0.2092 | 0.9033 |  |

**B)**

| **Least Squares Means for Effect of Eptifibatide Dose** | | | | | | | |
| --- | --- | --- | --- | --- | --- | --- | --- |
| **Dose (µM)** | **0** | **0.24** | **0.48** | **0.72** | **0.96** | **1.2** | **2.4** |
| **0** |  | <.0001 | <.0001 | <.0001 | <.0001 | <.0001 | <.0001 |
| **0.24** | <.0001 |  | 0.0004 | <.0001 | <.0001 | <.0001 | 0.004 |
| **0.48** | <.0001 | 0.0004 |  | 0.9969 | 0.8297 | 0.9369 | 1 |
| **0.72** | <.0001 | <.0001 | 0.9969 |  | 0.9862 | 0.9992 | 0.9999 |
| **0.96** | <.0001 | <.0001 | 0.8297 | 0.9862 |  | 0.9999 | 0.9643 |
| **1.2** | <.0001 | <.0001 | 0.9369 | 0.9992 | 0.9999 |  | 0.9929 |
| **2.4** | <.0001 | 0.004 | 1 | 0.9999 | 0.9643 | 0.9929 |  |
